# The Polycistronic miR166k-166h Positively Regulates Rice Immunity via Post-transcriptional Control of *EIN2*

**DOI:** 10.3389/fpls.2018.00337

**Published:** 2018-03-20

**Authors:** Raquel Salvador-Guirao, Yue-ie Hsing, Blanca San Segundo

**Affiliations:** ^1^Centre for Research in Agricultural Genomics CSIC-IRTA-UAB-UB, Universitat Autònoma de Barcelona, Barcelona, Spain; ^2^Institute of Plant and Microbial Biology, Academia Sinica, Taipei, Taiwan; ^3^Consejo Superior de Investigaciones Científicas, Barcelona, Spain

**Keywords:** blast, *ethylene-insensitive 2* (*EIN2*), miR166, *Oryza sativa*, *Magnaporthe oryzae*, rice

## Abstract

MicroRNAs (miRNAs) are small RNAs acting as regulators of gene expression at the post-transcriptional level. In plants, most miRNAs are generated from independent transcriptional units, and only a few polycistronic miRNAs have been described. miR166 is a conserved miRNA in plants targeting the *HD-ZIP III* transcription factor genes. Here, we show that a polycistronic miRNA comprising two miR166 family members, miR166k and miR166h, functions as a positive regulator of rice immunity. Rice plants with activated *MIR166k-166h* expression showed enhanced resistance to infection by the fungal pathogens *Magnaporthe oryzae* and *Fusarium fujikuroi*, the causal agents of the rice blast and bakanae disease, respectively. Disease resistance in rice plants with activated *MIR166k-166h* expression was associated with a stronger expression of defense responses during pathogen infection. Stronger induction of *MIR166k-166h* expression occurred in resistant but not susceptible rice cultivars. Notably, the *ethylene-insensitive 2* (*EIN2*) gene was identified as a novel target gene for miR166k. The regulatory role of the miR166h-166k polycistron on the newly identified target gene results from the activity of the miR166k-5p specie generated from the miR166k-166h precursor. Collectively, our findings support a role for miR166k-5p in rice immunity by controlling *EIN2* expression. Because rice blast is one of the most destructive diseases of cultivated rice worldwide, unraveling miR166k-166h-mediated mechanisms underlying blast resistance could ultimately help in designing appropriate strategies for rice protection.

## Introduction

MicroRNAs (miRNAs) are endogenous, small non-coding RNAs that mediate post-transcriptional gene silencing in eukaryotes (Jones-Rhoades et al., 2006). They are transcribed as long primary transcripts (pri-miRNAs), forming an imperfect fold-back structure, and are sequentially processed by a DICER-like ribonuclease (typically DCL1) to produce a pre-miRNA and finally a double-stranded miRNA duplex, the miRNA-5p/miRNA-3p duplex (previously named miRNA/miRNA^∗^ duplex) (Kurihara and Watanabe, 2004). The miRNA-5p/miRNA-3p duplexes are then transported to the cytoplasm, where the functional miRNA strand is incorporated into an ARGONAUTE1 (AGO1)-containing RNA-induced silencing complex (RISC) (Baumberger and Baulcombe, 2005; Jones-Rhoades et al., 2006; Rogers and Chen, 2013). miRNAs guide post-transcriptional gene silencing via sequence-specific cleavage or translational repression of target transcripts (Llave et al., 2002; Brodersen et al., 2008).

The crucial role of miRNAs in controlling plant developmental processes and response to abiotic stress is well documented (De Lima et al., 2012). Alterations in the accumulation of a substantial fraction of the miRNAome during pathogen infection is also described in different pathosystems, and for some miRNAs a role in plant immunity has been described (Shivaprasad et al., 2012; Campo et al., 2013; Boccara et al., 2014; Li et al., 2014; Baldrich and San Segundo, 2016; Soto-Suárez et al., 2017). However, our current knowledge of the biological roles of pathogen-regulated miRNAs in plant immunity is still limited, and most comes from studies in the interaction of *Arabidopsis thaliana* with the bacterial pathogen *Pseudomonas syringae* (Staiger et al., 2013; Weiberg et al., 2014; Fei et al., 2016; Kuan et al., 2016).

miRNAs are thought to have originated by duplication of pre-existing protein-coding genes with subsequent mutations (Allen et al., 2004; Rajagopalan et al., 2006). The spontaneous evolution from hairpin structures in the genome, or derivation from transposable elements, has also been proposed to explain the origin of plant miRNAs (Felippes et al., 2008; Nozawa et al., 2012). Whole-genome duplication events, and tandem or segmental duplications of *MIR* genes, are believed to be responsible for the expansion and diversification of miRNA gene families in plants (Maher et al., 2006; Nozawa et al., 2012). In animals, the occurrence of miRNA clusters is common, but only a few miRNA clusters have been described in plants, mainly in Arabidopsis (Boualem et al., 2008; Merchan et al., 2009; Barik et al., 2014; Baldrich et al., 2016). These clustered miRNAs can be transcribed independently or simultaneously as polycistronic transcripts. Furthermore, transcripts of polycistronic miRNAs might contain copies of members belonging to the same miRNA family (homologous polycistron), or unrelated miRNAs (non-homologous polycistron).

The miR166 family comprises multiple members in monocotyledonous and dicotyledonous plants that are transcribed independently (monocistrons). This is a highly conserved family of miRNAs with conserved target genes, the Class III homeodomain-leucine zipper (*HD-ZIP III*) transcription factors. These transcription factors, such as the Arabidopsis *PHABULOSA* (*PHB*) and *PHABOLUTA* (*PHV*), are involved in diverse developmental processes (Emery et al., 2003; Itoh et al., 2008). Altered accumulation of miR166 during abiotic stress also led to the notion that miR166 might play a role in the plant response to diverse abiotic stresses. Very recently, it has been described that miR166 knockdown triggers drought resistance in rice (Zhang et al., 2018). Evidence for miR166 in adapting to pathogen infection in plants has not been reported.

Recently, we described the occurrence of a rice polycistronic miRNA, miR166k-166h, comprising two miR166 family members (miR166k and miR166h). Expression profiling revealed that mature miRNAs generated from the miR166k-166h precursor are co-expressed in rice leaves (Baldrich et al., 2016). In other studies, various miR166 species were found to differentially respond to infection by the rice blast fungus *M. oryzae* or to differentially accumulate in blast-resistant and blast-susceptible rice varieties (Li et al., 2014, 2016).

In this work, we present evidence supporting that *MIR166k-166h* plays a role in rice immunity. We show that rice plants with activated *MIR166k-166h* expression exhibit resistance to infection by the fungal pathogens *M. oryzae* and *Fusarium fujikuroi*, the causal agents of the rice blast and bakanae disease, respectively. Rice blast is one of the most devastating diseases of cultivated rice due to its widespread distribution and destructiveness (Wilson and Talbot, 2009). The phenotype of disease resistance is associated with a stronger induction of defense responses during pathogen infection. *MIR166h-166k* expression was strongly induced by *M. oryzae* infection in blast-resistant but not in blast-susceptible rice varieties. Moreover, we identified a novel target gene for miR166k, the *ethylene-insensitive 2* (*EIN2*) gene (targeted by miR166k-5p in the miR166k-166h polycistron). Overall, our results support that the polycistronic miR166k-166h positively regulates rice immunity through modulation of *EIN2* expression.

## Materials and Methods

### Plant Material

Rice (*Oryza sativa*) plants were grown at 28°C/22°C under 16-h light/8-h dark conditions. The T-DNA insertion line for *MIR166k-166h* (M0110144) and wild-type genotype (*O. sativa japonica* cv Taining 67) were obtained from the Taiwan Rice Insertional Mutant collection (TRIM^1^). Genotyping of the TRIM mutant was carried out by PCR on genomic DNA using a T-DNA-specific primer located at the left border of the T-DNA and a primer located in the vicinity of the insertion site. PCR products were confirmed by DNA sequencing. Quantitative PCR (qPCR) was used to determine the T-DNA copy number in the rice mutant with the monocopy *sucrose phosphate synthase gene* used as the endogenous reference (Ding et al., 2004) (primers are listed in Supplementary Table S1).

The rice cultivars Saber, TeQing, Kanto 51, Maratelli and Vialone Nano were obtained from the germplasm seed bank of the Consiglio per la Ricerca e la Sperimentazione in Agricoltura (CRA-Rice Research Unit, Vercelli, Italy).

### Infection Assays and Elicitor Treatment

The fungus *M. oryzae* (strain *Guy-11*) was grown on complete medium as described (Campos-Soriano et al., 2012). For infection assays with *M. oryzae*, 3-week-old plants were spray-inoculated with a spore suspension (5 × 10^5^ spores/ml), or mock-inoculated. Development of disease symptoms was followed over time. Lesion area was determined by using Assess 2.0 software (American Phytopathological Society). For infection assays with *Fusarium fujikuroi*, the fungus was grown on PDA (Difco, Franklin Lakes, NJ, United States). Rice seeds were pregerminated for 24 h on Murashige and Skoog (MS) medium and then inoculated with a suspension of *F. fujikuroi* spores (1 × 10^6^ spores/ml), or sterile water. Seedlings were allowed to continue germination for 1 week. Three independent infection experiments were performed, with at least 24 plants per genotype in each experiment. Statistically significant differences were determined by one-way ANOVA. qPCR was used to quantify fungal DNA in infected leaves with specific primers for the 28S DNA gene of the corresponding fungus (Qi and Yang, 2002; Jeon et al., 2013). For this, standard curves were prepared by using *M. oryzae* or *F. fujikuroi* DNA.

For elicitor treatment, 3-week-old plants were sprayed with an elicitor suspension of *M. oryzae* (3 × 10^2^ μg/ml) or mock-inoculated as described (Casacuberta et al., 1992).

### 1-Aminocyclopropane-1-Carboxylic Acid (ACC) Treatment

Three-week old rice plants were treated with ACC (Merck, Darmstadt, Germany) at a concentration of 50 μM for 15 min, 1, 4, and 24 h. Control plants were mock-inoculated.

### RT-qPCR, Stem-Loop RT-PCR and 5′ RACE-PCR

Total RNA was extracted by using TRIzol Reagent (Invitrogen). First-strand cDNA was synthesized from DNAse-treated total RNA (1 μg) with SuperScript III reverse transcriptase (Invitrogen, Carlsbad, CA, United States) and oligo-dT. RT-qPCR was performed with Light Cycler 480 and SYBR Green (Roche, Basel, Switzerland). Primers were designed by using the Primer3 software^2^. Primers for detection of pre-miR166k-166h were designed based on the precursor sequence information from miRBase. PCR products were confirmed by DNA sequencing. The average cycle threshold (Ct) values were obtained by PCR from three independent biological replicates and normalized to the mean Ct values for *cyclophilin 2* gene (Os02g02890) from the same RNA preparations, yielding the relative expression (ΔCt value). The 2-ΔΔCt method was used to determine the fold-change of gene expression (infected/elicitor-treated “versus” mock-inoculated).

Stem-loop RT-qPCR was performed as described (Varkonyi-Gasic et al., 2007). Modified 5′-RNA ligase-mediated RACE was performed as described (Llave et al., 2011). The PCR products were cloned and sequenced to determine the cleavage site in target genes. Primers used for RT-qPCR and stem-loop RT-PCR are in Supplementary Table S1.

### Agroinfiltration in *Nicotiana benthamiana* Leaves

For transient expression of *MIR166k-166h*, the genomic DNA fragment encompassing the entire miR166k-166h precursor was obtained by PCR from genomic DNA and cloned into the pCAMBIA5300 vector (pC5300)^3^ under the control of the maize ubiquitin promoter. The *OsEIN2.1* cDNA sequence (Ma et al., 2013) was cloned into the pCAMBIA2300 expression vector (pC2300) designed to produce C-terminal GFP-tagged fusion proteins under the control of the 35S Cauliflower Mosaic Virus promoter. Plasmid constructs were introduced into the *Agrobacterium tumefaciens* EHA105 strain. As a negative control, the empty vector was used. The *N. benthamiana RDR6-IR* line deficient in expression of RNA-dependent RNA polymerase 6 was used as a host plant (Schwach et al., 2005). Constructs harboring the miR166k-166h precursor or *EIN2-GFP*, alone or in combination, were agroinfiltrated in *N. benthamiana* leaves, and their expression was monitored at 2 days after agroinfiltration.

Northern blot analysis of the agroinfiltrated leaves involved using the small RNA fraction obtained from total RNA (200 μg). The oligonucleotide complementary to the miR166 sequence (Supplementary Table S1) was labeled with digoxigenin with the DIG oligonucleotide 3′-End Labeling kit (Roche, Basel, Switzerland). For detection of the EIN2-GFP protein, total protein extracts (50 μg) were subjected to SDS-PAGE (12.5% gels) and probed with an anti-GFP antibody (Invitrogen, Carlsbad, CA, United States).

## Results

### *MIR166k-166h* Activation Enhances Resistance to Infection by the Rice Blast Fungus *M. oryzae*

**FIGURE 1:**
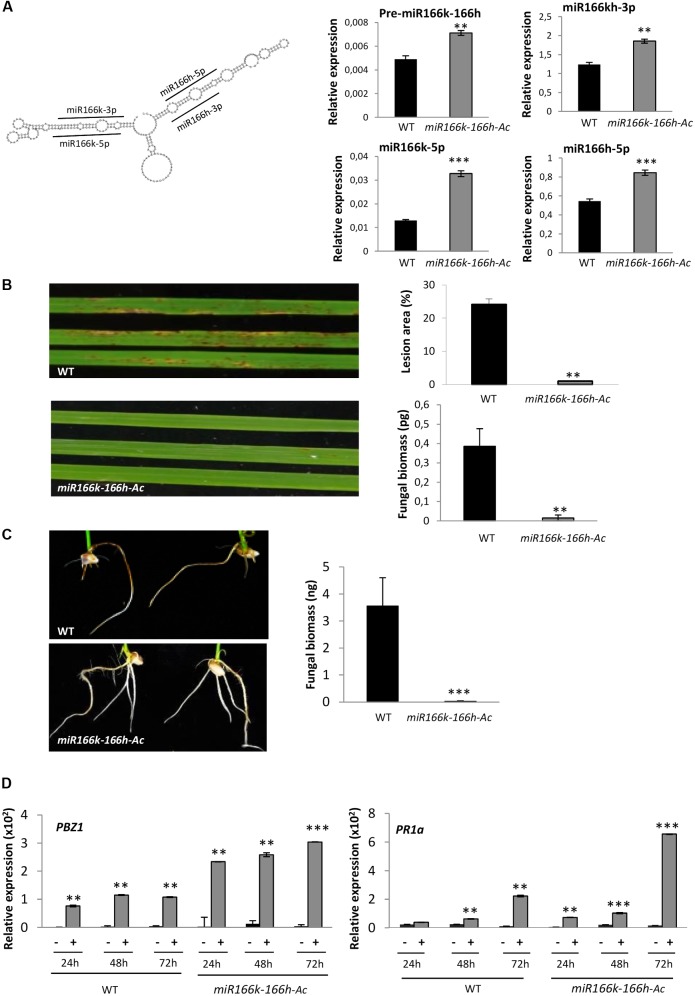
Characterization of polycistronic *miR166k-166h* mutant plants. **(A)** Structure of the miR166k-166h precursor and location of mature miR166 sequences (left panel). The accumulation of miR166k-166h precursor transcripts and mature miR166 sequences in wild-type (TN67) and *miR166k-166h-Ac* mutant plants was determined by RT-qPCR and stem-loop RT-qPCR, respectively (right panels). Note that the stem-loop RT-qPCR does not discriminate among miR166k-3p and miR166h-3p sequences (indicated as miR166kh-3p). **(B)** Phenotype of wild-type and *miR166k-166-Ac* mutant plants at 7 days post-inoculation with *M. oryzae* spores (5 × 10^5^ spores/ml). The percentage of leaf area affected by blast lesions was determined by image analysis (APS Assess 2.0) (right upper panel). Quantification of *M. oryzae* DNA was determined by qPCR with specific primers of the *M. oryzae* 28S gene (right lower panel). **(C)** Resistance to infection by *F. fujikuroi* in *miR166k-166h-Ac* mutant plants. Pictures were taken at 7 days after inoculation with fungal spores. Quantification of fungal DNA was carried out by qPCR using specific primers for *F. fujikuroi* (right panel). **(D)** Accumulation of transcripts for the defense marker genes *OsPBZ1* and *OsPR1a* in wild-type and *miR166k-166h-Ac* plants in response to *M. oryzae* infection determined by RT-qPCR. Plants were inoculated with *M. oryzae* spores (5 × 10^5^ spores/ml) or mock-inoculated (+ and –, respectively). Ct values obtained in the PCR reactions were normalized to the average Ct values for the *cyclophilin 2* gene (for graphical representation, the values are multiplied by 100). Data are mean ± SD (^∗∗∗^*P* ≤ 0.001; ^∗∗^*P* ≤ 0.01, ANOVA test, *M. oryzae*-inoculated *versus* mock-inoculated).

The rice genome contains several loci encoding monocistronic miR166s distributed on 7 chromosomes: miR166a, miR166b, miR166c, miR166d, miR166e, miR166f, miR166g, miR166i, miR166j, miR166l and miR166m (miRBase release 21) (Supplementary Figure S1). Furthermore, a polycistronic miR166 encoding two miR166 family members, the miR166k-166h precursor, was identified on chromosome 2 (Baldrich et al., 2016). The mature miR166k and miR166h species locate in one or another hairpin of the miR166k-166h precursor structure (**Figure 1A**, left panel). Of note, loci encoding monocistronic transcripts for miR166k or miR166h have not been identified in the rice genome.

In this work, a T-DNA tagged line (M0110144) carrying the T-DNA insertion upstream of the *MIR166k-166h* locus was identified in the TRIM collection produced in the Tainung 67 (TN67) background (Hsing et al., 2007). Of note, mutant alleles for miRNAs are not easily found in insertional mutant collections because of the small size of *MIR* genes. The T-DNA contains 8 copies of the *CaMV35* enhancer near the left border, and genes within 15 kb of the T-DNA left border and 5 kb of the right border might be activated by these enhancers. Knowing this, we hypothesized that this mutant might be an activation mutant for *MIR166k-166h*. The T-DNA insertion site was confirmed by PCR genotyping followed by DNA sequencing of the PCR products (Supplementary Figure S2A). No homozygous *MIR166k-166h* plants were identified in PCR genotype screens. Most importantly, heterozygous mutant plants accumulated higher levels of miR166k-166h precursor transcripts, which correlated well with an increase in the accumulation of mature miR166k and miR166h sequences (**Figure 1A**, right panel). Since the rice genome does not contain monocistronic miR166k and miR166h loci, the miR166k and miR166h mature sequences accumulating in rice leaves are expected to be generated from the polycistronic miR166k-166h precursor. These observations confirmed that the TRIM mutant is an activation mutant for *MIR166k-166h* (hereafter referred to as *miR166k-166h-Ac*). However, miR166 has been shown to repress the seed maturation program in Arabidopsis, and difficulties in generating transgenic lines overexpressing miR166 were previously reported (Tang et al., 2012). Presumably, high levels of miR166 expression and concomitant silencing of *HD-ZIP III* might compromise normal plant development. Therefore, it is not surprising that homozygous *miR166k-166h-Ac* mutant plants could not be identified in this study. The *miR166k-166h-Ac* mutant harbors a single copy of the T-DNA inserted in its genome (Supplementary Table S2).

We considered the possibility that the expression of genes other than *MIR166k-166h* might be activated in the *miR166k-166h-Ac* mutant. Two genes, *OsSAUR12* (Os02g52990) and *Erwinia-induced protein* (Os02g53000), were identified upstream and downstream, respectively, of the T-DNA insertional site (Supplementary Figure S2A). However, we found no altered accumulation of *OsSAUR12* or *Erwinia-induced protein* transcripts in the *miR166k-166h-Ac* mutant (Supplementary Figure S2B). There were no obvious phenotypic differences between miR166k-166h mutant and wild-type plants under controlled greenhouse conditions (Supplementary Figure S2C).

To investigate whether miR166k-166h miRNA plays a role in rice immunity, we performed blast disease resistance assays. Wild-type (cv TN67) and *miR166k-166h-Ac* plants were spray-inoculated with spores of the rice blast fungus *M. oryzae*, and disease symptoms were followed over time. The *miR166k-166h-Ac* plants consistently showed reduced disease symptoms as compared with wild-type plants (**Figure 1B**, left panel). Blast resistance was confirmed by quantification of fungal biomass and determination of lesion area in the infected leaves (**Figure 1B**, right panels).

The *miR166k-166h-Ac* mutant plants also showed enhanced resistance to infection by the fungus *F. fujikuroi*, the causal agent of bakanae in rice (Ou, 1985). The fungus infects the plant through the roots (or crowns) and grows systemically within the plant. At 7 days after inoculation, the *miR166k-166h-Ac* seedlings exhibited more vigorous growth of the root system compared to wild-type seedlings which also had extensive necrosis in their roots (**Figure 1C**, left panel). Quantification of fungal biomass confirmed limited fungal growth in roots of *miR166k-166h-Ac* seedlings (**Figure 1C**, right panel).

To obtain further insights into the mechanisms underlying disease resistance in the *miR166k-166h-Ac* mutant, we determined the expression pattern of the defense genes *OsPBZ1* (*Probenazole-inducible 1*) and *OsPR1a* (*Pathogenesis-Related 1a*) in mutant and wild-type plants at different times after infection with *M. oryzae* (24, 48, and 72 h post-inoculation [hpi]). *OsPBZ1* (a member of the *PR10* family of *PR* genes) and *OsPR1a* genes are markers for the activation of the rice defense response to *M. oryzae* infection (Midoh and Iwata, 1996; Agrawal et al., 2001). As expected, fungal infection induced *OsPR1a* and Os*PBZ1* expression in wild-type plants. Importantly, transcript levels of these defense genes were higher in *M. oryzae*-inoculated *miR166k-166h-Ac* than *M. oryzae*-inoculated wild-type plants at all times of infection (**Figure 1D**). These findings support that the *miR166k-166h-Ac* mutant responds to pathogen challenge with a super-induction of defense genes, which is consistent with the phenotype of disease resistance observed in these plants.

### *MIR166k-166h* Expression During Fungal Infection and Treatment With Elicitors

**FIGURE 2:**
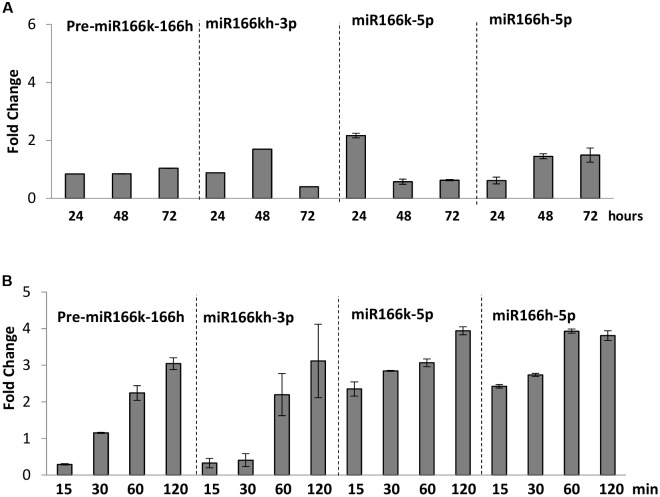
*MIR166k-166h* expression in response to *M. oryzae* infection or treatment with *M. oryzae* elicitors in wild-type (cv. TN67) plants. The accumulation of miR166k-166h precursor and mature miR166 sequences was determined by RT-qPCR and stem-loop RT-qPCR, respectively, at different times after inoculation with *M. oryzae* spores (5 × 10^5^ spores/ml) **(A)** or treatment with *M. oryzae* elicitors (3 × 10^2^ μg/ml) **(B)**. Values represent the fold-induction of gene expression (Ct) in *M. oryzae*-inoculated *versus* mock-inoculated plants. Three independent experiments were carried out (*P* ≤ 0.05, ANOVA test).

Given that activation of *MIR166k-166h* affects disease resistance, we sought to investigate whether *MIR166k-166h* expression is itself regulated during the normal host response to infection. Upon pathogen challenge, miR166k-166h precursor transcript level was increased in leaves of *M. oryzae*-inoculated compared with non-inoculated wild-type TN67 plants, with a parallel increase in mature miR166k and miR166h sequences (both miRNA-5p and miRNA-3p species) (**Figure 2A**). Interestingly, accumulation of precursor and mature miR166 sequences also increased in response to treatment with a crude preparation of elicitors (**Figure 2B**). Elicitor treatment resulted in faster induction of miR166k-5p and miR166h-5p species *versus* miR166kh species. Induction of marker genes of defense activation, *OsPBZ1* and *OsPR1a*, confirmed that the host plant detects and responds to elicitor treatment (Supplementary Figure S3A). Finally, we examined the elicitor-responsiveness of the monocistronic miR166s, miR166a and miR166c. The accumulation of precursor transcripts for these miR166 family members (pre-miR166a and pre-miR166c) was found to be transiently, but not significantly, regulated during elicitor treatment (Supplementary Figure S3B).

From these results, we concluded that pathogen infection and also treatment with fungal elicitors upregulates *MIR166k-166h* expression, which suggests a role of this polycistronic miRNA in pathogen-associated molecular pattern (PAMP)-triggered immunity (PTI).

The promoter region of protein-coding genes often includes *cis*-acting regulatory elements responsible for pathogen inducibility. Knowing that fungal infection and elicitor treatment induced *MIR166k-166h* expression, we scanned the *MIR166k-166h* promoter region for the presence of *cis*-regulatory elements related to biotic stress. The sequence upstream of the precursor structure for the miR166k-166h precursor was extracted from the NCBI database and the transcription start site (TSS) was identified by using the TSSP Softberry program for identifying TSS in plants^4^. *Cis*-acting elements present in the 1.6 Kb DNA region upstream of the TSS were searched in the PLACE database^5^. The *MIR166k-166h* promoter was found to contain an important number of *cis*-elements required for response to pathogen infection or elicitor treatment (Supplementary Figure S4 and Supplementary Table S3). We identified several W-boxes (TGAC core sequences), such as WBOXATNPR1 (TTGAC), elicitor responsive element (ERE; TTCAGG), WRKY710S (TGAC), WBOXNTERF3 (TGACY), and ASF1 (TGACG) *cis*-elements (Supplementary Figure S4). These regulatory *cis*-elements are the binding sites for salicylic acid-induced WRKY transcription factors and are also found in many pathogen- and elicitor-responsive genes. The SEBF regulatory element (SEBFCONSSTPR10A, YTGTCWC), initially characterized in the promoter of the pathogen and elicitor inducible potato *PR-10a* gene and later in the promoter of several other *PR* genes, was also identified in the *MIR166k-166h* promoter. Other functional pathogen/elicitor-responsive elements identified were the GT1-SCAM4 (GAAAAA) and PAL-responsive (CCGTCC) elements. Finally, regulatory elements associated with defense-related hormone signaling also present in the *MIR166k-166h* promoter included the ethylene (ERELEE4, ethylene-responsive element; AWTTCAAA) and methyl jasmonic acid (T/G BOXPIN2, AACGTG) regulatory elements. Although pathogen/hormone-responsive *cis*-elements were identified in the *MIR166k-166h* promoter, their functionality in controlling *MIR166k-166h* expression remains unknown.

### *MIR166k-166h* Expression in Resistant and Susceptible Rice Varieties

**FIGURE 3:**
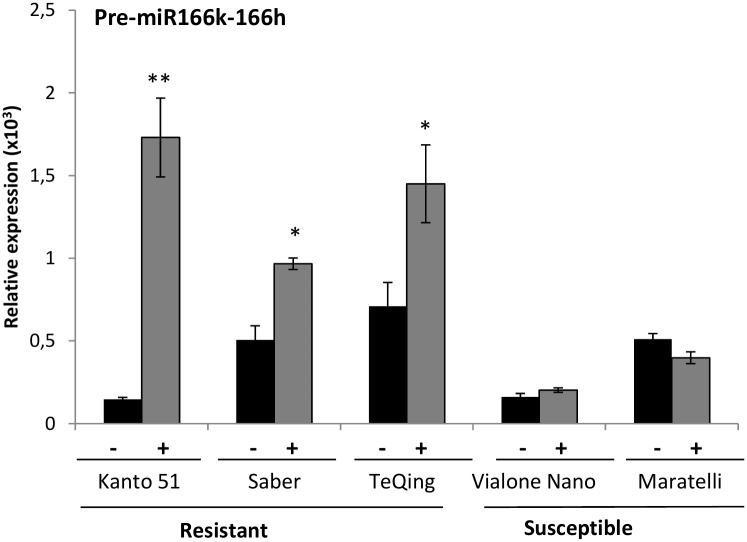
Accumulation of miR166k-166h precursor transcripts in susceptible and resistant rice varieties *M. oryzae* spores or mock-inoculated (+ and –, respectively). RT-qPCR analysis of accumulation of miR166k-166h precursor transcripts at 72 hpi. Data are mean ± SD (^∗∗^*P* ≤ 0.01; ^∗^*P* ≤ 0.05, ANOVA test).

We examined *MIR166k-166h* expression in rice varieties showing a phenotype of disease resistance against the rice blast fungus: Kanto 51, Saber and TeQing (resistant varieties), and Vialone Nano and Maratelli (susceptible varieties). The resistant genotypes are characterized by the presence of the resistance (R) genes: *Pik* in Kanto51, and P*ib* in Saber and TeQing (Tacconi et al., 2010). The basal level of expression varied among the different rice varieties (**Figure 3**). At 72 hpi with *M. oryzae*. *MIR166k-166h* expression was strongly induced in the three resistant rice genotypes here assayed, whereas its expression was barely affected or was even decreased by *M. oryzae* infection in the susceptible cultivars Vialone Nano and Maratelli (**Figure 3**). Thus, induction of *MIR166k-166h* expression appears to occur in resistant but not susceptible rice cultivars.

### Prediction and Experimental Validation of a Novel Target for miR166

**FIGURE 4:**
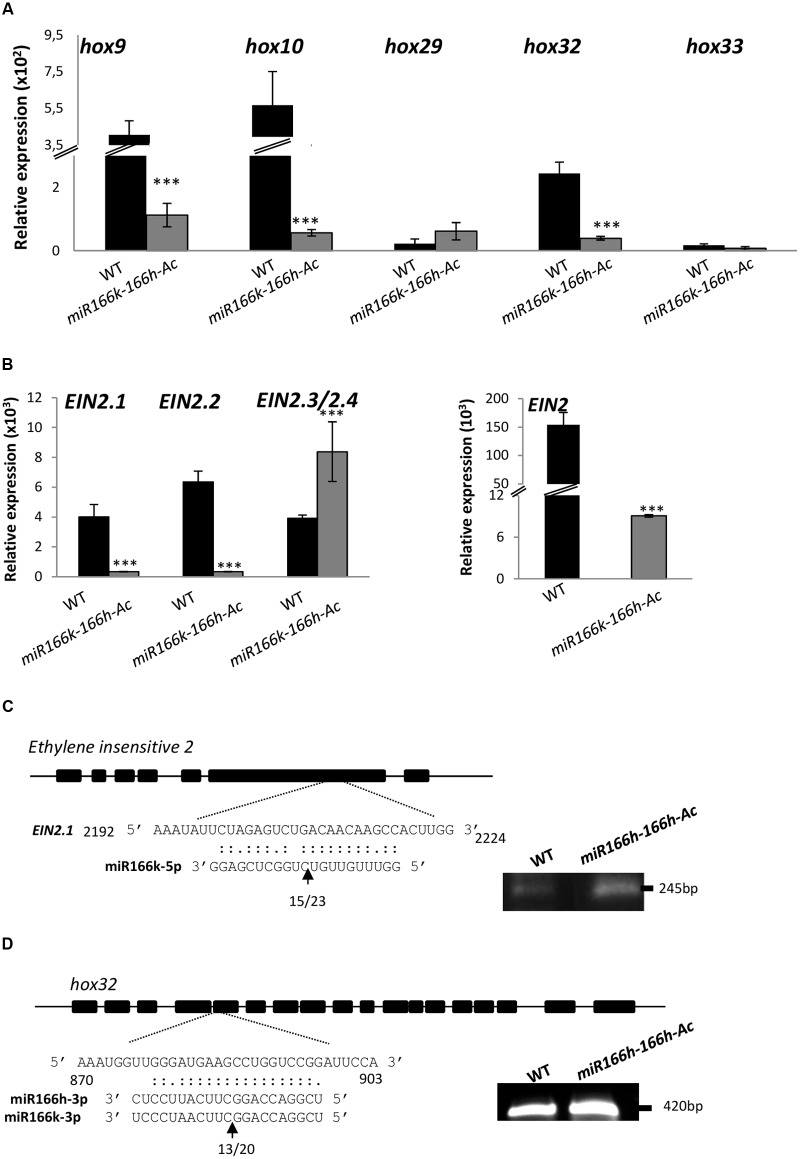
Expression of miR166 targets and identification of a novel target gene for miR166. **(A)** RT-qPCR analysis of accumulation of *Oshox* transcripts encoding HD-ZIP III transcription factors in wild-type and *miR166k-166h-Ac* plants. **(B)** Expression of *OsEIN2* genes. Left panel: Gene-specific PCR primers were used for quantification of *OsEIN2.1, OsEIN2.2* or *OsEIN2.3/2.4* transcripts (*EIN2.3 and EIN2.4* sequences are closely related each other, and specific primers for these genes could not be designed). Right panel: RT-PCR using primers spanning the miR166 target site in *EIN2* transcripts. Data (A, B) are mean ± SD (^∗∗∗^*P* ≤ 0.001; ^∗∗^*P* ≤ 0.01, ANOVA test). **(C,D)** Experimental validation of miR166-mediated cleavage of *OsEIN2.1* **(C)** and *Oshox32* **(D)** transcripts by 5′ RACE. Gene-specific primers were used for 5′ RACE and the resulting PCR products (right panels) were sequenced. The identified cleavage sites are indicated by an arrow, and the numbers below indicate the detected cleavage site of independent clones. WT, TN67. Data are mean ± SD (^∗∗∗^*P* ≤ 0.001; ^∗∗^*P* ≤ 0.01, ANOVA test).

As previously mentioned, *HD-ZIP III* genes are conserved target genes for miR166 in plants. In monocistronic miR166s, the mature miR166 sequences that direct cleavage of *HD-ZIP III* transcripts are located at the 3′ arm of the precursor structure, namely miR166h-3p and miR166k-3p. In rice, five *HD-ZIP III* genes have been described: *Oshox9* (Os10g33960), *Oshox10* (Os03g01890), *Oshox29* (Os01g10320), *Oshox32* (Os03g43930) and *Oshox33* (Os12g41860) (Agalou et al., 2008). Degradation tags indicative of miR166-mediated cleavage of *Oshox9*, *Oshox10*, *Oshox32*, and *Oshox33* were identified by degradome analysis, which supports that they are real targets of rice miR166s (Li et al., 2010; Baldrich et al., 2015). In addition, RT-qPCR analysis revealed reduced levels of *Oshox9*, *Oshox10*, and *Oshox32* in *miR166k-166h-Ac* mutant versus wild-type plants (**Figure 4A**), which confirms the functionality of mature miRNAs encoded by the polycistron. As for *Oshox29* and *Oshox33*, these genes were found expressed at very low levels in wild-type plants, and their expression was not significantly affected in *miR166k-166h-Ac* mutant plants as compared with wild-type plants (**Figure 4A**).

Knowing that *MIR166k-166h* activation has an impact on blast resistance, we considered the possibility that this phenotype might be caused by the activity of miR166 species encoded in the miR166k-166h precursor on novel, non-conserved target genes. We performed a target prediction analysis by using the psRNATarget tool^6^. Similar to other species, the target search predicted *HD-ZIP III* as target genes of miR166s encoded in the miR166k-166h polycistron (miR166k-3p and miR166h-3p). This computational prediction identified a putative target gene for the miR166k-5p sequence, the *EIN2* gene. As for miR166h-5p, a possible binding site for this miRNA in a *ferredoxin-nitrite-reductase* gene was predicted.

A function for *EIN2* as mediator of ethylene-dependent defense responses in plants is well established (Iwai et al., 2006; Helliwell et al., 2013, 2016; Yang et al., 2017). Accordingly, in this work we investigated whether *EIN2* is a target gene for miR166k-5p. In contrast to Arabidopsis, in which EIN2 is encoded by a single gene, the rice genome possesses four *EIN2* genes: *OsEIN2.1* (also named *MHZ7*; Os07g06130), *OsEIN2.2* (Os03g49400), *OsEIN2.3* (Os07g06300), and *OsEIN2.4* (Os07g06190) (Ma et al., 2013; Yang et al., 2015). Based on sequence homology, *OsEIN2* genes can be classified into two groups, the first comprising *OsEIN2.1* and *OsEIN2.2* and the second *OsEIN2.3* and *OsEIN2.4*. The four *OsEIN2* genes have the binding site for miR166k-5p (Supplementary Figure S5A). RT-qPCR analysis was used to quantify *OsEIN2.1, OsEIN2.2* and *OsEIN2.3/4* expression in wild-type (TN67) and mutant plants (with the high sequence homology between *OsEIN2.3* and *OsEIN2.4*, we could not design PCR-specific primers for these genes). Location of the primers used for detection of *EIN2.1*, *EIN2.2* and *EIN2.3/2.4* is shown in Supplementary Figure S5B. This analysis revealed downregulation of *OsEIN2.1* and *OsEIN2.2* in *miR166k-166h-Ac* plants (**Figure 4B**, left panel). The observed inverse correlation between mature miR166k-5p levels and *EIN2.1* and *EIN2.2* transcripts in *miR166k-166h-Ac* plants already indicated a possible miR166k-5p-mediated downregulation of this particular *OsEIN2* family members. Intriguingly, *OsEIN2.3/4* transcripts accumulated to a higher level in *miR166k-166h-Ac* mutant than wild-type plants. The amount of uncleaved *OsEIN2* transcripts was determined by using PCR primers flanking the miR166k-5p cleavage site. Although the accumulation of uncleaved *EIN2* transcripts was notably reduced in the activation mutant, uncleaved transcripts still accumulated to an important level in these plants, likely due to the contribution of *EIN2.3/EIN2.4* transcripts (**Figure 4B**, right panel). This observation suggests the existence of complex regulatory mechanisms governing the expression of *OsEIN2* in rice in which downregulation of *OsEIN2.1* and *OsEIN2.2* expression is accompanied by an increase in *EIN2.3/EIN2.4* transcripts.

These observations prompted us to further investigate whether *EIN2* gene is a real target gene for miR166k-166h by performing RNA ligase-mediated 5′ RACE (5′-RACE). Sequencing of the 5′-RACE PCR clones revealed cleavage fragments of *EIN2.1* transcripts (**Figure 4C**, left panels). Transcripts were found cleaved at the canonical position of miRNA/target mRNA pairing (between nucleotides 10 and 11 from the 5′ end of the miRNA), which supports that *EIN2* is indeed a target gene for miR166 in rice. As well, cleavage products of *EIN2.1* accumulated to a lower level in wild-type than *miR166k-166h-Ac* plants (**Figure 4C**, right panel). As a control, miR166-guided cleavage products of *hox32* were also identified by 5’-RACE (**Figure 4D**). Altogether, these results demonstrated that miR166 cleaves *EIN2.1* transcripts and that the miR166k-5p strand in the miR166k-166h precursor is functional.

### miR166k-166h Mediates Cleavage of *EIN2* Transcripts That Reduce Levels of *EIN2* Protein

Among the newly identified miR166 targets, *EIN2* is worth describing specifically. This gene is a central signal transducer in the ethylene signaling pathway in plants, and ethylene signaling is known to modulate plant immune responses (Solano and Ecker, 1998; Jun et al., 2004; Denancé et al., 2013; Ma et al., 2013).

**FIGURE 5:**
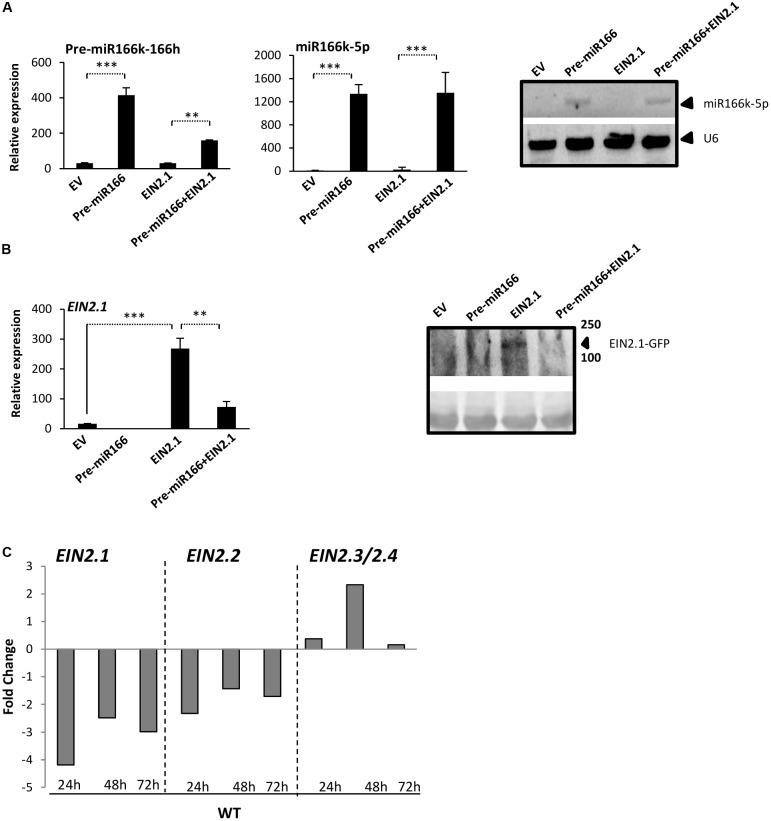
Validation of miR166k-166h-mediated downregulation of Os*EIN2.1* in agroinfiltrated *N. benthamiana* leaves **(A,B)** and *OsEIN2* expression in wild-type rice plants during *M. oryzae* infection **(C)**. **(A,B)** Agroinfiltration of *N. benthamiana* leaves with constructs harboring the miR166k-166h precursor only (pre-miR166), the *EIN2.1-GFP* only (EIN2), or the miR166k-166h precursor and *EIN2.1-GFP* (pre-miR166+EIN2). The empty vector (EV) was a control. Expression analyses were performed 2 days after agroinfiltration. **(A)** Accumulation of miR166k-166h precursor and miR166k-5p sequences determined by RT-qPCR and ST-RT-qPCR, respectively (left and middle panels). Detection of mature miR166 sequences by small-RNA Northern blot analysis is shown on the right panel. For Northern blot analysis, the small RNA fraction of agroinfiltrated leaf samples was hybridized with a 3′ end digoxigenin-labeled oligonucleotide sequence complementary to miR166k-5p. The same blot was stripped and rehybridized with the U6 probe. **(B)** Accumulation of *OsEIN2.1* transcripts and EIN2 protein in agroinfiltrated *N. benthamiana* leaves (left and right panels, respectively). Western blot analysis (right panel) with polyclonal anti-GFP antibody. Ponceau staining of the protein blot is shown in the bottom panel. **(C)** *EIN2* expression in wild-type (cv TN67) plants in response to *M. oryzae* infection. Data are fold-change repression (*EIN2.1*, *EIN2.2*) or induction (*EIN2.3/2.4*) of gene expression (*M. oryzae*-inoculated “versus” mock-inoculated) at the indicated times after inoculation with *M. oryzae* spores. Data are mean ± SD (^∗∗∗^*P* ≤ 0.001; ^∗∗^*P* ≤ 0.01, ANOVA test).

To further investigate the functional relationship between miR166h-166k activity and *EIN2* expression, we performed agroinfiltration experiments in *N. benthamiana* leaves in which the miR166k-166h precursor and a GFP-tagged *EIN2.1* gene were co-expressed. As controls, constructs bearing the empty vector, the miR166k-166h precursor alone, or the *EIN2.1-GFP* chimeric gene alone were also assayed in agroinfiltration experiments. RT-PCR analysis revealed the accumulation of precursor miR166k-166h transcripts in agroinfiltrated leaves (**Figure 5A**, left panel). Accumulation of mature miR166 sequences derived from this precursor was confirmed by ST-RT-qPCR and Northern blot analyses (**Figure 5A** and Supplementary Figure S6). These analyses indicated that the miR166k-166h precursor is properly expressed and processed in *N. benthamiana* leaves when expressed alone or with *EIN2.1-GFP*. However, levels of miR166k-166h transcripts were higher in miR166k-166h-only agroinfiltrated leaves *versus* leaves in which the miR166k-166h precursor was co-expressed with *EIN2.1* (**Figure 5A**, left panel, pre-miR166 and pre-miR166+*EIN2*), an aspect that deserves further investigation.

When examining the transcript accumulation of *EIN2.1*, co-expression of the miR166k-166h precursor with *EIN2.1*-*GFP* reduced the *EIN2.1*-*GFP* transcript level as compared with expression of *EIN2.1-GFP* alone (**Figure 5B**, left panel, *EIN2* and pre-miR166+*EIN2*). The observed reduction in *EIN2.1-GFP* transcripts was accompanied by a reduced EIN2-GFP protein level, as revealed by immunoblotting of protein extracts with an anti-GFP antibody (**Figure 5B**, right panel). From these results, we conclude that miR166k-166h targets and cleaves *OsEIN2.1* and that cleavage of *OsEIN2.1* transcripts reduces EIN2 protein accumulation.

Finally, knowing that *MIR166k-166h* expression is upregulated during *M. oryzae* infection in wild-type (cv TN67) plants (**Figure 2A**), and that *OsEIN2.1* is a target gene for miR166, we investigated the expression of *OsEIN2* family members during pathogen infection. *OsEIN2.1* and *OsEIN2.2* were downregulated during *M. oryzae* infection (**Figure 5C**), which is consistent with the observed increase in miR166k-5p level in the same tissues. In contrast, *OsEIN2.3/4* expression was upregulated during pathogen infection. Presumably, the increased level of miR166k-5p in *M. oryzae*-infected leaves would be responsible for downregulation of *OsEIN2.1* during pathogen infection.

### Expression of Ethylene Signaling Components in *miR166k-166h-Ac* Plants

In the absence of ethylene, phosphorylation of EIN2 prevents transduction of ethylene signaling. However, in the presence of ethylene, EIN2 phosphorylation is reduced and the C-terminal fragment of EIN2 is cleaved and translocated to the nucleus where the downstream EIN3 and EIL1 transcriptional cascade is activated. In addition, EIN2 and EIN3/EIL1 are regulated by proteasomal degradation via EIN3-binding F-box protein 1 and 2 (EBF1/2). Then, EIN3 and EIL1 regulate the expression of ethylene-responsive genes including *Ethylene Response Factor 1* (*ERF1*) which, in turn, modulates the expression of various ethylene-responsive genes such as *PDF1.2* and *chitinase* genes (Lorenzo et al., 2003; Abiri et al., 2017). It is generally assumed that *EIN2* functions as a positive regulator of ethylene signaling, as revealed by repression of ethylene-inducible defense genes in *ein2* antisense rice plants (Jun et al., 2004). The construct used to obtain *ein2* antisense rice plants covered a 638-bp DNA fragment of the *EIN2.1* cDNA encompassing the C-terminal region of *EIN2*, a region with high sequence conservation among *OsEIN2* family members. Thus, silencing of all four *OsEIN2* genes is expected to occur in the *ein2* antisense plants previously described (Jun et al., 2004).

Accumulating evidence also indicates that ethylene signaling is required in rice for basal resistance against the blast fungus *M. oryzae* (Singh et al., 2004; Iwai et al., 2006; Helliwell et al., 2013, 2016; Yang et al., 2017). Thus, the observed increase in miR166k-166h accumulation and concomitant downregulation of *OsEIN2.1* and *OsEIN2.2* expression in *miR166k-166h-Ac* plants (**Figures 1**, **4**, respectively) apparently contradicts *OsEIN2* positively regulating ethylene signaling in the rice response to *M. oryzae* infection.

**FIGURE 6:**
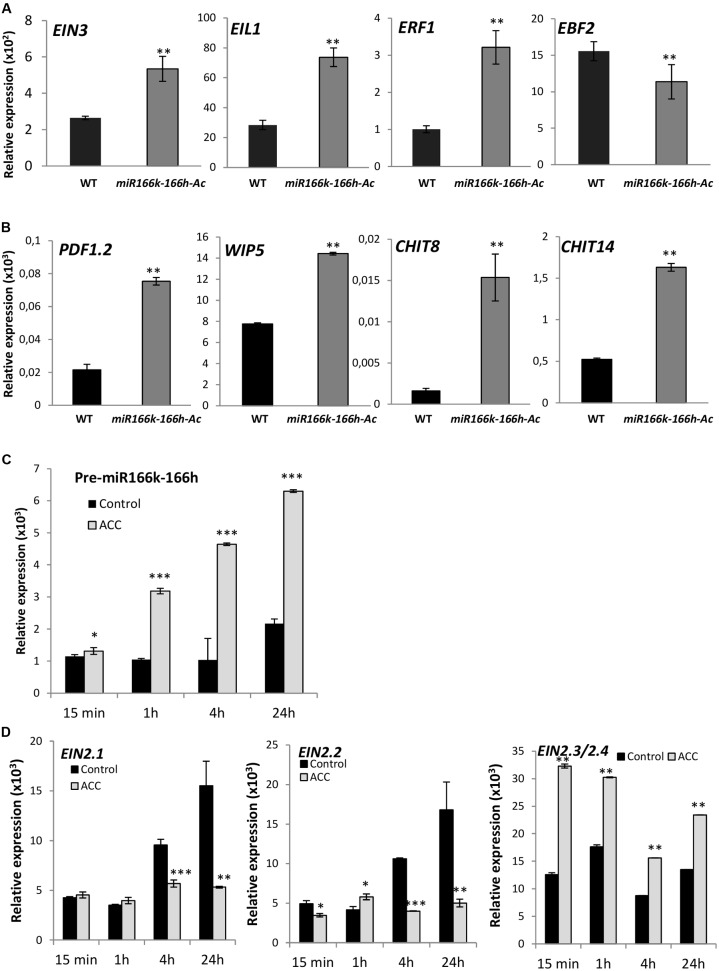
Expression of ethylene signaling components in *miR166k-166h-Ac* mutant plants, and *MIR166k-166h* expression in response to treatment with the ethylene precursor ACC. RT-qPCR analysis of the accumulation of transcripts for the indicated genes with use of gene-specific PCR primers. Ct values obtained in the PCR reactions were normalized to the average Ct values for the *cyclophilin 2* gene. **(A)** Expression of genes acting downstream of *EIN2* (*OsEIN3*, *OsEIL1*, *OsERF1* and *OsEBF2*) in wild-type and *miR166k-166h-Ac* plants. **(B)** Expression of *OsPDF1.2*, *OsWIP5* (*PR4* family), *OsCHIT8* and *OsCHIT14* (*PR3* family). **(C)** Accumulation of miR166k-166h precursor transcripts in control and ACC-treated (50 μM) wild-type plants. **(D)** Accumulation of *EIN2.1*, *EIN2.2* and *EIN2.3/2.4* in control and ACC-treated wild-type plants. Data are mean ± SD (^∗∗∗^*P* ≤ 0.001; ^∗∗^*P* ≤ 0.01; ^∗^*P* ≤ 0.05; ANOVA test).

To address the apparent contradiction of downregulation of *OsEIN2* expression in *miR166h-166k-Ac* plants, showing blast resistance, we investigated the expression of genes acting downstream of *EIN2* in the ethylene signaling pathway in mutant plants. *OsEIN3* and *OsEIL1*, as well as *OsERF1*, were upregulated in *miR166k-166h-Ac* plants as compared with wild-type plants, whereas *OsEBF2* expression was downregulated (**Figure 6A**). Consistent with upregulation of *OsERF1*, the expression of ethylene-responsive defense genes, such as *PDF1.2* and *chitinase* genes (e.g., *CHIT8* and *CHIT14*, members of the *PR3* family of *PR* genes; and *WIP5*, a *PR4* family member) was also upregulated in *miR166k-166h-Ac* plants (**Figure 6B**). These data indicate that although miR166k-166h activation downregulates *OsEIN2.1* and *OsEIN2.2*, components in the pathway for ethylene signal transduction downstream of *OsEIN2* are induced in *miR166k-166h-Ac* plants, which would agree with the resistance phenotype that is observed in *miR166k-166h-Ac* mutant plants. Knowing that *OsEIN2.3/2.4* is activated in the *miR166k-166h-Ac* mutant (see **Figure 4B**), *OsEIN2.3/2.4* activation is likely responsible for the observed induction of downstream components of ethylene signaling in these plants, including ethylene-regulated defense genes.

To provide additional clues for the function of miR166k-166h in rice immunity, we investigated whether *MIR166k-166h* expression itself is regulated by ethylene in wild-type plants. For this, wild-type plants were treated with the ethylene precursor ACC, and the accumulation of miR166k-166h precursor transcripts was determined at different times after ACC treatment (15 min, 1, 4, and 24 h). ACC treatment resulted in a clear and gradual increase in the accumulation of miR166k-166h precursor transcripts in wild-type plants (**Figure 6C**).

Finally, expression analysis were performed to determine the accumulation of *EIN2* transcripts in wild-type in response to ACC treatment. Consistent with up-regulation of *MIR166k-166h* in response to ACC treatment, *EIN2.1* and *EIN2.2* were found to be down-regulated during the same period of treatment (**Figure 6D**). However, EIN2.3/2.4 transcripts accumulated at a higher level in ACC-treated plants compared to control plants (**Figure 6D**). Thus, a different trend in the regulation of *EIN2* family members occurs in response to ACC treatment which correlates with differences previously observed between *miR166k-166h-Ac* mutant plants and wild-type plants (see **Figure 4B**).

## Discussion

In this work, we provide evidence that the polycistronic miR166k-166h plays a role in rice immunity. Thus, activation of *MIR166k-166h* in *miR166k-166h-Ac* plants, and concomitant increase in mature miR166s derived from the miR116h-166k precursor, enhances resistance to infection by hemibiotrophic (*M. oryzae*) and necrotrophic (*F. fujikuroi*) fungal pathogens (Ou, 1985; Wilson and Talbot, 2009; Campos-Soriano et al., 2013). Resistance to *M. oryzae* infection in *miR166k-166h-Ac* plants is associated with a stronger induction of defense gene expression, at both the biotrophic (24–48 hpi) and necrotrophic (72 hpi) stages of the infection. In wild-type plants, miR166k-166h accumulation was increased during pathogen infection and also in response to treatment with fungal elicitors, which supports that *MIR166k-166h* is a component of PTI. The observation that *MIR166k-166h* expression is activated in resistant rice cultivars but not in susceptible varieties (72 hpi with *M. oryzae* spores) further supports the role of *MIR166k-166h* in the rice response to the rice blast fungus. A more detailed analysis is, however, needed to examine the expression kinetics of *MIR166k-166h* in resistant and susceptible rice varieties during the infection process.

Of note, *MIR166k-166h* is found in the genome of both *japonica* and *indica* subspecies of the *O. sativa* genus (AA genome) (Baldrich et al., 2016). The *MIR166k-166h* locus is also present in the genome of wild relatives of current cultivated rice varieties, namely *O. rufipogon* and *O. nivara* (wild relatives of *O. sativa*), and *O. barthii* (wild relative of *O. glaberrima*, or African rice). These observations support conservation of the miR166k-166h polycistron in the *Oryza* genus (Baldrich et al., 2016). miR166 clusters have been identified in the genome of several plant species (e.g., *M. truncatula*, soybean and *Physcomitrella patens*) but, in most cases, the polycistronic nature of these miR166 clusters has not been demonstrated (Boualem et al., 2008; Zhang et al., 2009; Barik et al., 2014; Li et al., 2017).

Our evidence supports that *EIN2* is a novel target gene for miR166, this gene being targeted by miR166k-5p in the *MIR166k-166h* polycistron. Supporting this conclusion, we found opposite expression patterns of miR166k-5p and *OsEIN2.1* in *miR166k-166h-Ac* mutant plants. Also, miR166k-5p and its target gene showed opposite expression patterns in response to fungal infection (upregulation and downregulation, respectively). Definitive proof of a miR166k-5p-mediated cleavage of *EIN2.1* transcripts came from 5′-RACE analyses and agroinfiltration experiments in *N. benthamiana* leaves. The observed miR166-guided cleavage of *EIN2.1* transcripts was accompanied by reduced EIN2 protein level. From these results, we conclude that *EIN2.1* represents a novel target gene for miR166k-5p species encoded by the polycistronic miR166k-166h precursor.

Clearly, the existence of multiple miR166 family members might contribute to diversification and functional specialization of miR166 in plants. In line with this, miR166b has been reported to target rice *RDD1* (*rice Dof daily fluctuations 1*), a non-*HD-ZIP III* transcription factor involved in nutrient uptake and accumulation (Iwamoto and Tagiri, 2016). Very recently, miR166-guided cleavage of *ATHB14-LIKE* transcripts encoding a homeobox-leucine zipper protein has been described in soybean (Li et al., 2017). In *M. truncatula*, a miR166 polycistron containing two copies of miR166a targeting *HD-ZIP III* transcripts was found to control root architecture and nodule development after infection by *Sinorhizobium meliloti* (Boualem et al., 2008). Presumably, mature miRNAs encoded by the miR166k-166h polycistron might have evolved to mediate rice defense responses to pathogen infection.

When considering the mature miR166s encoded by the miR166k-166h precursor, we noticed that miR166 species targeting *OsEIN2.1* correspond to miR166-5p in monocistronic miR166s, while miR166-3p sequences target *hox* genes. Hence, the two strands of the miR166k duplex in the miR166k-166h precursor appear to be functional. There are other examples in which the two strands of a miRNA are functional, as for miR393 in Arabidopsis. Here, the miR393 strand guides cleavage of transcripts encoding auxin receptor genes (*TIR1*, *AFB2*, *AFB3*), and the miR393-3p strand cleaves *MEMB12* transcripts encoding a SNARE protein involved in exocytosis of the PR1 protein (Zhang et al., 2011). Degradome analysis revealed miR166e-3p and miR166h-5p-mediated events for genes involved in the arbuscular mycorrhizal symbiosis in *Medicago truncatula* (e.g., *Sucrose synthase*, *Tyr protein kinase* and *protein phosphatase 2C*) (Devers et al., 2011). In addition to being represented by multiple copies in the rice genome, the ability of miR166 precursors to produce two mature functional strands in the same miRNA-5p/miRNA-3p duplex also represents an effective strategy to diversify miR166 function.

Our results indicate that *MIR166k-166h* activation enhances defense gene expression, most probably by modulating *OsEIN2* expression. An intriguing aspect of this study was the finding of a different trend in the regulation of *OsEIN2* expression in *miR166k-166h-Ac* plants depending on the family member. Whereas *EIN2.1* and *EIN2.2* are downregulated in the rice mutant (the two genes being more closely related to one another than either *EIN2.3* or *EIN2.4*), *EIN2.3* and *EIN2.4* are upregulated in these plants. Additional regulatory forces controlling the abundance of *EIN2* transcripts must then exist. Several possibilities can be considered to explain the finding of *OsEIN2.1* and *OsEIN2.2* being downregulated in *miR166k-166h-Ac* plants and *OsEIN2.3/2.4* upregulated in this mutant. They include the existence of regulatory mechanisms in which miR166k-5p and *EIN2* family members regulate each other’s expression, or interconnecting networks controlling the expression of *OsEIN2* family members themselves (i.e., the abundance of a particular *OsEIN2* gene might affect the level of another *OsEIN2* family member). Previous studies in Arabidopsis demonstrated cross-regulation among transcription factor family members targeted by miRNAs (i.e., regulation of GROWTH REGULATING FACTORS by miR396 species) (Hewezi and Baum, 2012). Cross-regulation of auxin response factors (ARFs) has been also described, where ARF6 and ARF8 (targets of miR167) and ARF17 (targets of miR160) regulate each other’s expression at both transcriptional and posttranscriptional levels by modulating miR160 and miR167 availability (Gutierrez et al., 2009). The possibility of miR166k-5p-mediated translational repression of *EIN2* family members should not be ruled out. If such regulatory mechanisms operate in rice, this would represent an additional layer of regulation of *OsEIN2* expression, which would help in maintaining appropriate *OsEIN2* levels, rather than completely turning off *OsEIN2* expression to allow optimal expression of defense responses with no negative impact on plant growth.

**FIGURE 7:**
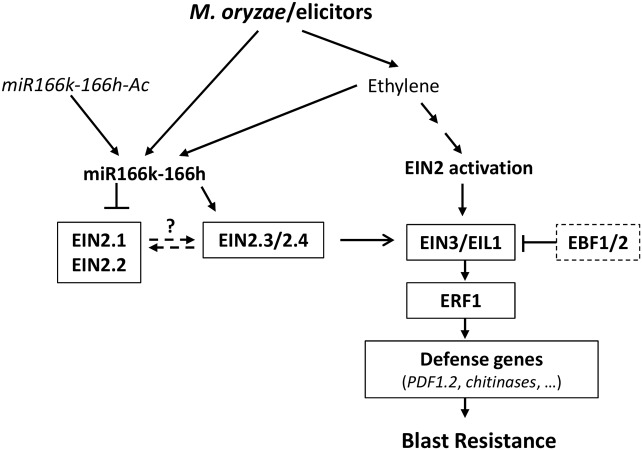
Proposed model for the function of miR166k-166h in ethylene signaling during infection of rice plants by the blast fungus *M. oryzae*. In the absence of ethylene, active ethylene receptors negatively regulate OsEIN2 via phosphorylation, thus repressing the downstream signaling transduction. Pathogen recognition would trigger ethylene biosynthesis, which is perceived by its receptors. Upon ethylene perception, the EIN2 phosphorylation is reduced and the carboxy-terminal fragment of EIN2 is cleaved and translocated to the nucleus for activation of EIN3/ELI1 and Ethylene Response Factor 1 (ERF1), thereby activating defense gene expression. Pathogen-induced ethylene production would also induce *MIR166k-166h* expression, which would then regulate the expression of *OsEIN2* family members (downregulation of *OsEIN1.2* and *OsEIN2.2*; upregulation of *OsEIN2.3/2.4*). *MIR166k-166h* activation in *miR166k-166h* mutant plants would mimic the activation of ethylene signaling pathways induced by *M. oryzae* infection in the host plant. Arrows and blunt ends indicate positive and negative regulation, respectively. Arrows with broken lines indicate still unknown interlocked regulatory mechanisms among EIN2 family members.

A working model of the role of miR166k-166h in governing expression of ethylene-regulated defense genes is in **Figure 7**. According to this model, pathogen recognition triggers ethylene biosynthesis and activation of *MIR166k-166h* expression, which in turn would regulate components of the ethylene signaling pathway leading to induction of ethylene-regulated defense genes (*PDF1.2*, *chitinases*). We propose an interlocking regulation mechanism governing the expression of *OsEIN2* family members and downstream signaling components leading to activation of defense gene expression. Further studies are required to determine the interlocking mechanisms among *OsEIN2* family members and among miR166k-miR166h and *EIN2*.

Basal resistance to *M. oryzae* has been reported to require activation of ethylene biosynthesis and signaling networks during the biotrophic phase of the infection process (Singh et al., 2004; Iwai et al., 2006; Helliwell et al., 2013, 2016; Yang et al., 2017). However, the mechanisms by which the pathogen induces ethylene biosynthesis remain unknown. Because *MIR166k-166h* expression is itself regulated by treatment with the ethylene precursor ACC (**Figure 6**), the *M. oryzae*-induced production of ethylene might induce *MIR166k-166h* expression. Furthermore, the *M. oryzae*-mediated ethylene accumulation has been found to affect JA signaling (Yang et al., 2017). Whether defense hormone networking is altered in *miR166k-166k-Ac* plants deserves further investigation.

It is also known that ethylene has antagonistic effects in controlling the rice defense response depending on the pathogen lifestyle and also on the type of pathogen. Whereas the accumulation of ethylene appears to be required for resistance against *M. oryzae* (Iwai et al., 2006), repression of ethylene signaling has been shown to enhance resistance against the necrotrophic rice brown spot fungus *Cochliobolus miyabeanus* (Vleesschauwer et al., 2010). A major future challenge is to determine the molecular processes by which *MIR166k-166h* function is integrated in the complex regulatory mechanisms involved in ethylene-regulated immune responses to *M. oryzae* infection and whether activation of *MIR166h-166k* expression confers resistance to pathogens other than *M. oryzae*.

Besides playing a role in plant responses to pathogen infection, ethylene is considered a phytohormone involved in regulation of plant growth and development. Because excessive ethylene production under pathogen infection might negatively affect plant development, the host plant must then maintain a tight control of ethylene homeostasis to cope with pathogenic infections with no growth penalty. In this respect, negative feedback mechanisms have been proposed to coordinate plant growth and ethylene/salinity responses (Tao et al., 2015).

Given the well-established roles of miR166 and its *HD-ZIP III* target genes in controlling developmental processes in a broad range of plant species, an intriguing question is why *MIR166k-166h* activation does not affect normal growth in the *miR166k-166h* mutant. A possible threshold of miR166k-166h level (and subsequent miR166-regulated *Oshox* transcripts) might explain this observation. Under heterozygosity, the *miR166k-166h-Ac* mutant plants would not accumulate sufficient levels of miR166kh species to alter normal developmental programs due to excessive downregulation of miR166 *HD-ZIP III* target genes. Moderate levels of mature miR166s produced by the miR166k-166h polycistron would provide a means to mount a more successful defense response without no penalty on normal development.

The functional significance of the organization of miRNAs as polycistrons is still debated. Polycistronic transcription can fine-tune gene expression in related or unrelated biological processes (e.g., defense responses and developmental processes). A single promoter drives the expression of polycistronic miRNAs, which allows for the expression of multiple miRNAs in a coordinated spatial and/or temporal manner.

## Conclusion

Our results support that miR166k-166h is a positive regulator of rice immunity via regulation of *OsEIN2*. A better knowledge of miR166k-166h functioning in blast resistance will help in deciphering the functional consequences of polycistronic expression of miRNAs in plants. Because blast is one of the primary causes of rice losses worldwide, unraveling miR166k-166h-mediated mechanisms underlying blast resistance could ultimately help in designing novel strategies for crop protection.

## Author Contributions

RS-G performed the experiments and analyzed the data. Y-iH and BSS designed and conceived the work. All the authors contributed to the manuscript writing.

## Conflict of Interest Statement

The authors declare that the research was conducted in the absence of any commercial or financial relationships that could be construed as a potential conflict of interest.

## Footnotes

1 http://trim.sinica.edu.tw/

2 http://www.ncbi.nlm.nih.gov

3 www.cambia.org

4 http://softberry.com/

5 http://www.dna.affrc.go.jp/PLACE

6 http://plantgrn.noble.org/psRNATarget
