# Supplementary material for: The Polycistronic miR166k-166h Positively Regulates Rice Immunity via Post-transcriptional Control of EIN2
Source: Front Plant Sci. 2018 Mar 20;9:337. doi: 10.3389/fpls.2018.00337 (PMC5869255; doi:10.3389/fpls.2018.00337)
Supplement: Supplementary file 1 [file Image_1.PDF]

# *Supplementary Material*

**Polycistronic miR166k-166h positively regulates rice immunity via post-transcriptional control of *EIN2***

Raquel Salvador-Guirao[1], Yue-Ie Caroline Hsing[2] and Blanca San Segundo[1,3*]

*** Correspondence:** Blanca San Segundo: blanca.sansegundo@cragenomica.es

## Supplementary Figures

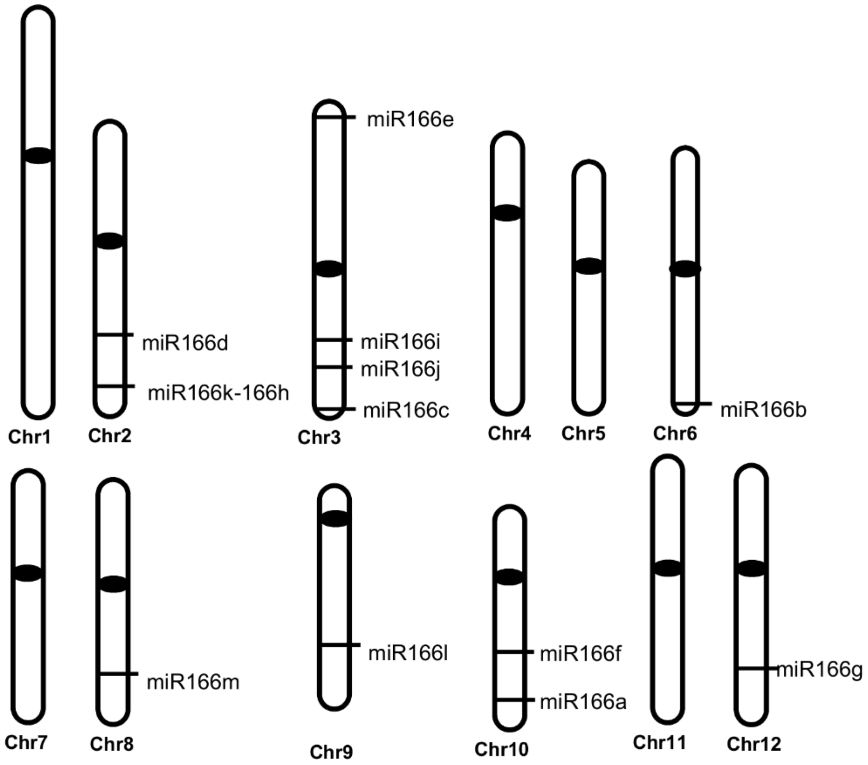


**Supplementary Figure S1.** Chromosomal location of monocistronic miR166s and polycistronic miR166k-166h in rice. The centromere in each chromosome is indicated by a black circle.

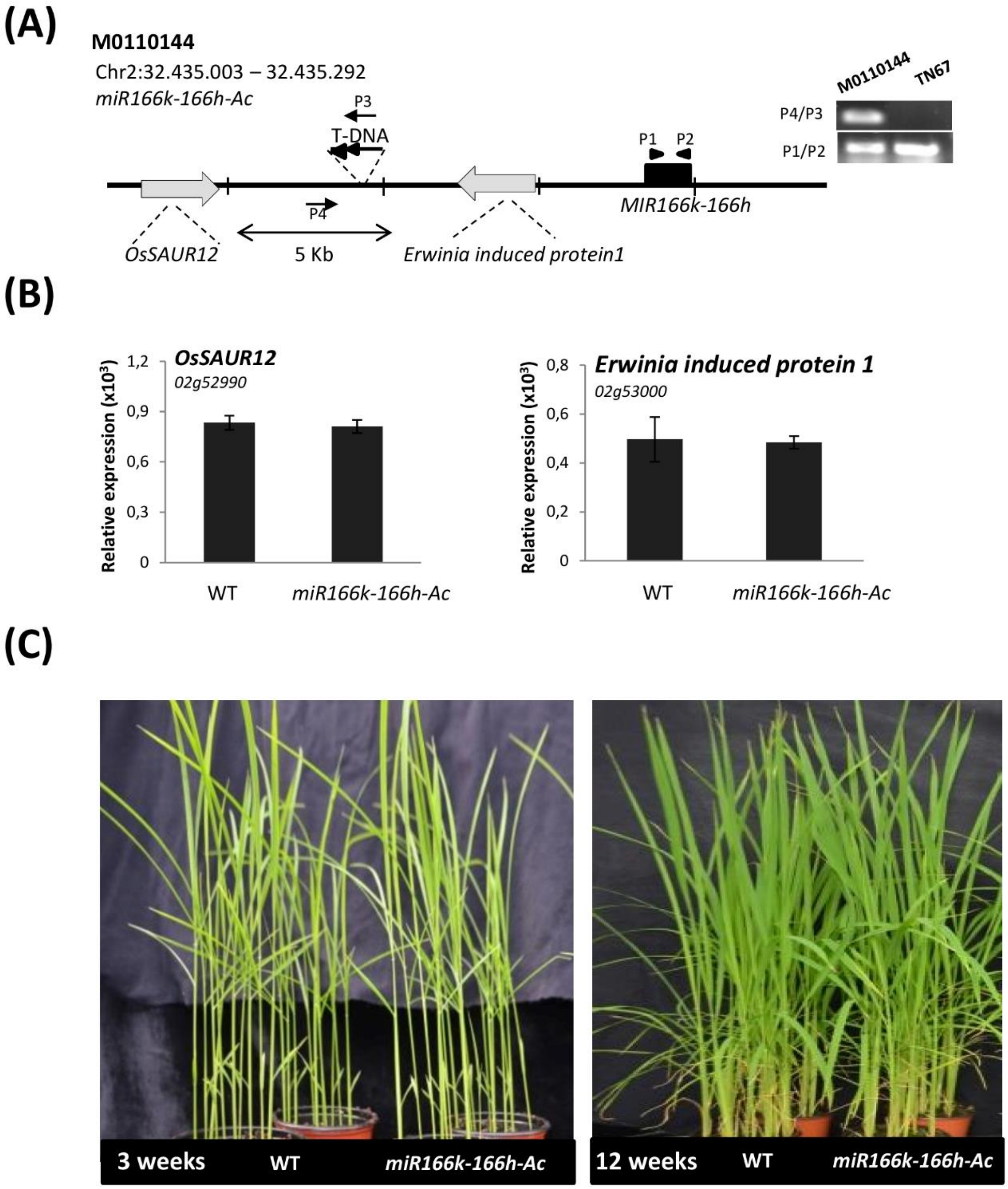


**Supplementary Figure S2.** Characterization of the *miR166k-166h-Ac* mutant (M0110144) from the TRIM collection**. (A)** Schematic representation of the T-DNA in the *miR166k-166h-Ac* mutant. PCR genotyping is shown in the right panel. Arrows and arrowheads indicate the position of primers used for genotyping. (**B)** Expression of genes flanking the T-DNA determined by RT-qPCR. Data are mean ± SD (ANOVA test). (**C)** Phenotype of wild-type and *miR166k-166h-Ac* plants at 3 and 12 weeks.

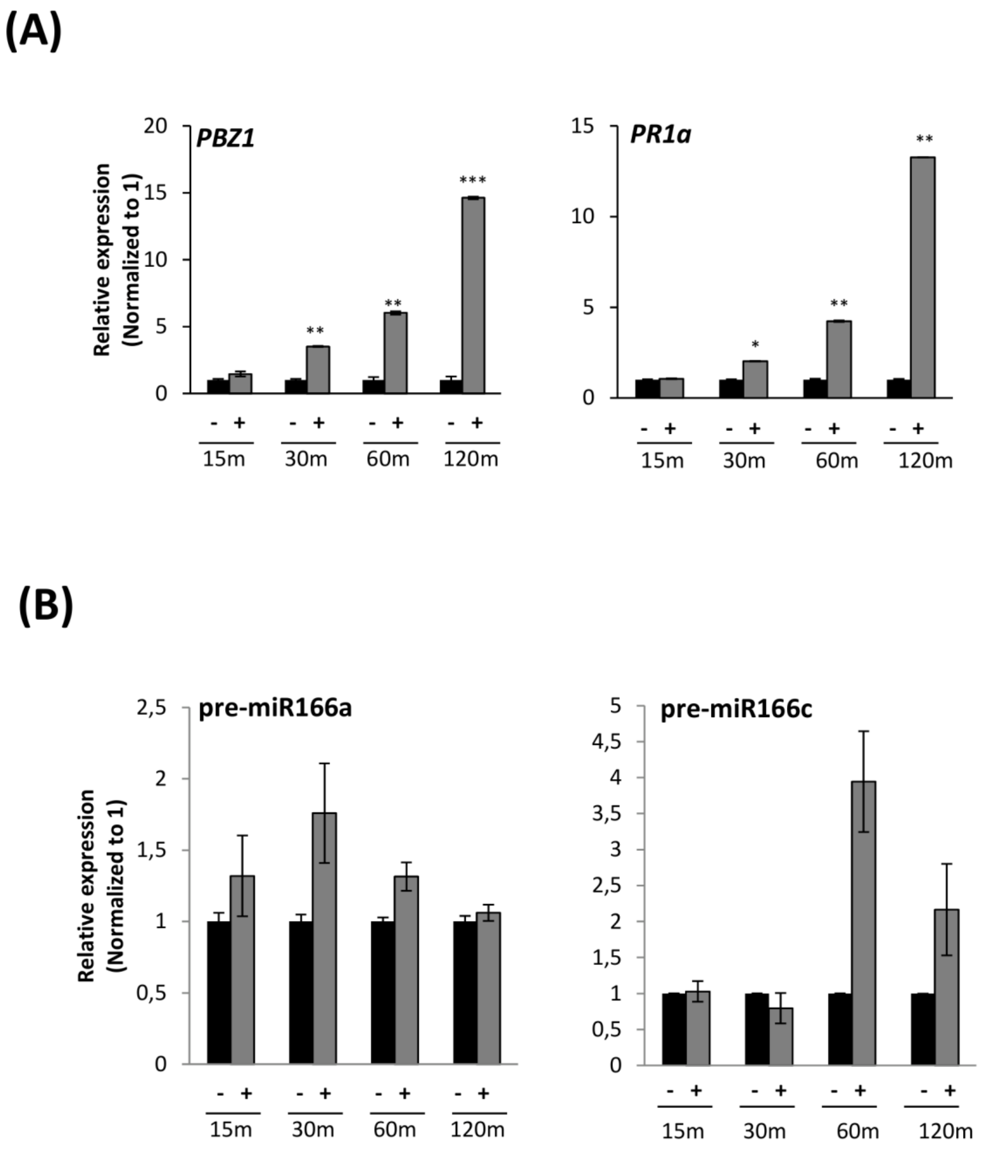


**Supplementary Figure S3.** (A) Accumulation of *OsPBZ1* and *OsPR1a* transcripts in wild-type (cv TN67) plants in response to treatment with *M. oryzae* elicitors. (B) Accumulation of pre-miR166a and pre-miR166c (monocistronic miR166 family members) in response to elicitors. RT-qPCR analysis was carried out in elicitor-treated and mock-inoculated plants at the indicated times after inoculation (+ and -, respectively). Relative expression is normalized to 1. Asterisks denote statistically significant differences (***, P ≤ 0.001; **, P ≤ 0.01, *, P ≤ 0.05, ANOVA test).

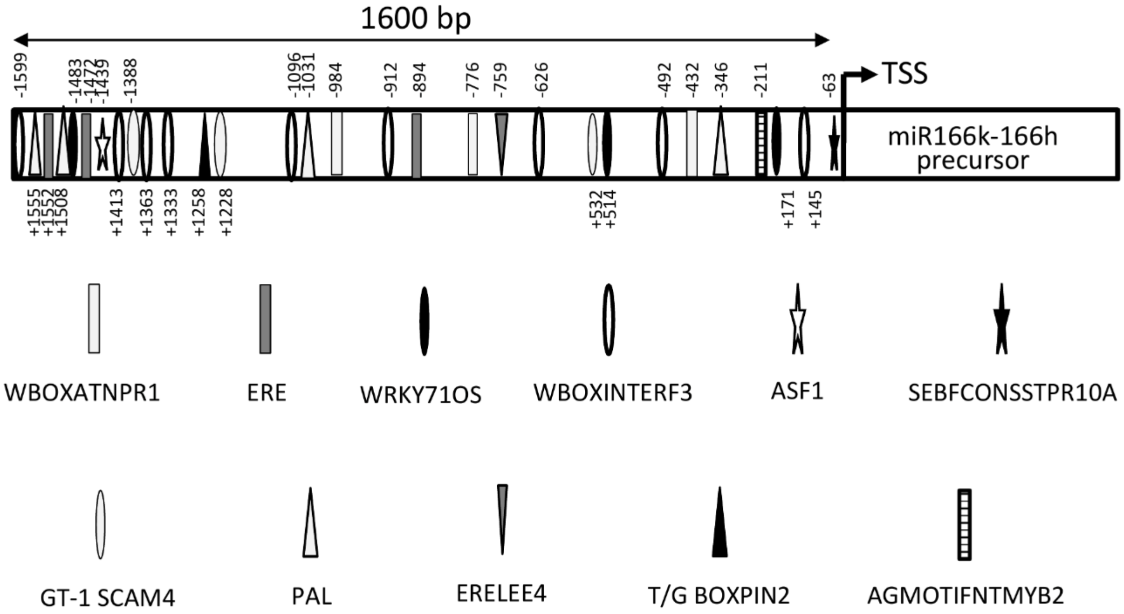


**Supplementary Figure S4.** Structural features of the *MIR166k-166h* promoter. The location of known *cis*-acting elements related to biotic stress is shown (for details on *cis*-elements, see **Table S3**). TSS, transcriptional start site.

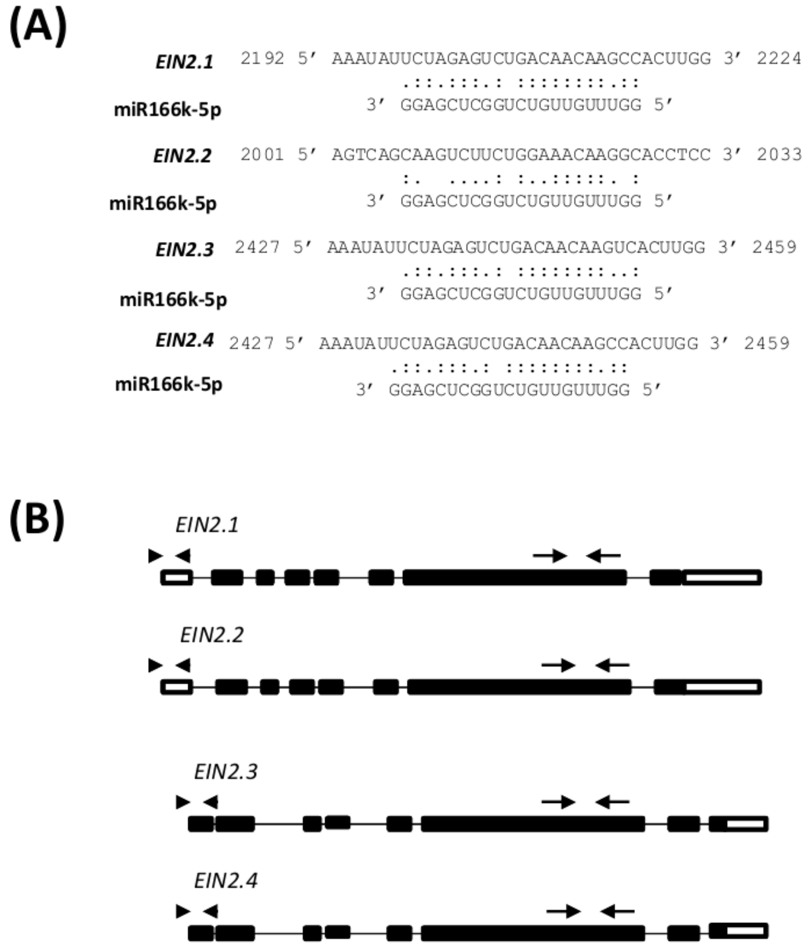


**Supplementary Figure S5. Sequence alignment of *EIN2* genes with miR166k-5p.** (A) The sequence corresponding to the binding site for miR166k-5p in the corresponding *EIN2* gene is shown. (B) Location of the primers used for detection of *EIN2* transcripts by RT-qPCR. Comparison of the nucleotide sequences of the four *EIN2* genes revealed differences in the 5'UTR region that allowed to design primers for specific detection of *EIN2.1*, *EIN2.2 and EIN2.3/2.4* (due to the high sequence similarity, specific primers for *EIN2.3* and *EIN2.4* could not be designed). The position of *EIN2*-specific and common primers for *EIN2* genes is indicated by arrowheads and arrows, respectively.

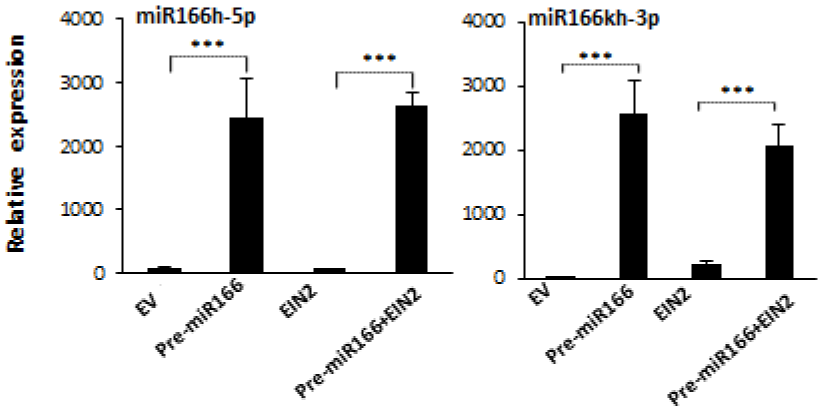


**Supplementary Figure S6.** Agroinfiltration assays in *N. benthamiana* leaves. Analysis of constructs harboring the miR166k-166h precursor only (pre-miR166), the *OsEIN2.1-GFP* only (EIN2), or the miR166k-166h precursor and *OsEIN2.1-GFP* (pre-miR166+*EIN2*). The empty vector (EV) was a control. Expression analyses were performed at 2 days after agroinfiltration. The accumulation of miR166k-166h precursor transcripts was determined by RT-qPCR, and accumulation of miR166h* and miR166 mature sequences was assayed by ST-RT-PCR. Data are mean ± SD (***, $P \leq 0.001$; ANOVA test).

**Supplementary Table S1.** Sequences of oligonucleotides used.

| Oligo ID | Accesion number | | Sequence (5'-3') |
|---|---|---|---|
| **For expression analysis** | | | |
| *Cyclophilin 2* | *LOC_Os02g02890* | *Frw* | GTGGTGTTAGTCTTTTTATGAGTTCGT |
| | | *Rev* | ACCAAACCATGGGCGATCT |
| pre-miR166k-166h | | *Frw* | CCTCGAGCCAGACAACAAAC |
| | | *Rev* | GGTGGCTTGTGGGGAATGTTGGCTGG |
| pre-miR166a | | *Frw* | CTCATGCACCTTGCGGTTTT |
| | | *Rev* | CGAGATCCCACGGATGAGAG |
| pre-miR166c | | *Frw* | CTGGTCCGAGACCTAACACC |
| | | *Rev* | GGAATGAAGCCTGGTCCGAA |
| *Erwinia induced protein 1* | *LOC_Os02g53000* | *Frw* | ATGGCGGGGGTGTGTGG |
| | | *Rev* | ACGACGACACCATCAC |
| *PR1a* | *LOC_Os07g03710* | *Frw* | CTGCAAGCTGGAGCACTCG |
| | | *Rev* | AAGATGTTCTCGCCGTACTTCC |
| *PBZ* | *LOC_Os12g36830* | *Frw* | GCGATGGCTCCTGTGTGG |
| | | *Rev* | CTCCGGCGACAGTGAGCT |
| *PDF1.2* | *LOC_OsOs02g12060* | *Frw* | GACGACGAGGAGGAGGACGA |
| | | *Rev* | ATGGAGCCTTCAGGCAAGCT |
| *PR3(CHIT8)* | *LOC_Os06g51060* | *Frw* | TCCCACCGCTTCTTAACATCA |
| | | *Rev* | ATGGTGGCAATGCAAAGCAAA |
| *PR3(CHIT14)* | *LOC_Os10g39680* | *Frw* | CTTCTACACGTACGACGCCTTCATCGCC |
| | | *Rev* | AGGTGCCGAACGCCGGGAA |
| *PR4(WIP5)* | *LOC_Os11g37970* | *Frw* | CTGCTGCACAAGAAGCATCC |
| | | *Rev* | CCCAGTTGTTCTGTTGTGGG |
| *EIN3* | *LOC_Os03g20780* | *Frw* | ATGGGAGGTGGTCTGGTGATGGA |
| | | *Rev* | CCACGAAGTTGTGCACGCC |
| *EIL1* | *LOC_Os03g20790* | *Frw* | TACAGATTGAAGAAGGCTTTTTT |
| | | *Rev* | AACACACCTCCGTTGCTGCCGCC |
| *ERF1* | *LOC_Os04g46220* | *Frw* | GAGTCGTCCTTCTCCTCCTC |
| | | *Rev* | CCTCTCTTTCTCCGTTTCG |
| *EBF2* | *LOC_Os06g40360* | *Frw* | TGGTGGTGTTCTTGTGATGG |
| | | *Rev* | TGACGCCGCCGAACAGAGCC |
| hox9 | *LOC_Os10g33960* | *Frw* | GGGACTGCTGTTGATTGGGTG |
| | | *Rev* | GCCACAATACCAAACGAAT |
| *hox10* | *LOC_Os03g01890* | *Frw* | ATTGATTGGGTCCAGATGCC |
| | | *Rev* | GCCACAATACCAACCGAAT |
| *hox29* | *LOC_Os01g10320* | *Frw* | GTGATCACAGGATGGGGAAGGC |
| | | *Rev* | CTCTGGTGAGCTTTTGGCTCAGA |
| *hox32* | *LOC_Os03g43930* | *Frw* | GTCGATTGGGTGCAAATGGT |
| | | *Rev* | TGTGCGAAACAGCGATGAT |
| *hox33* | *LOC_Os12g41860* | *Frw* | TGCTGTCGAATGGGTGCAAATGG |
| | | *Rev* | GAAACAGCGATGATTCCAATGGAAT |
| *EIN2* | | *Frw* | GATAAGGAGGCTCCAAATAT |
| | | *Rev* | CAATAATCCTTCCTGTGCAT |
| *EIN2.1* | *LOC_Os07g06130* | *Frw* | TGTTGGTGACTGAGAAGCCAT |
| | | *Rev* | ATGAGAAGTCCCTCTTGGGAA |
| *EIN2.2* | *LOC_Os03g49400* | *Frw* | AACGGCGTGTGTTTGGTTAAT |
| | | *Rev* | CGCGATACTACGCCTGGTC |
| *EIN2.3* | *LOC_Os07g06300* | *Frw* | TCTTTCTAGCTCCGCCATGC |
| *EIN2.4* | *LOC_Os07g06190* | *Rev* | ATGCATGTGTCAACCGAAGC |
| miR166k-5p | | *Frw* | GGCGGGGTTTGTTGTCTGG |
| | | *SL-RT* | GTCGTATCCAGTGCAGGGTCCGAGGTATTCGCACTGGATACGACCCTCGA |
| miR166h-5p | | *Frw* | GGCGGGGAATGTTGGCTGG |
| | | *SL-RT* | GTCGTATCCAGTGCAGGGTCCGAGGTATTCGCACTGGATACGACCCTCGA |
| miR166k-3p | | *Frw* | GGCGGTCGGACCAGGCTTC |
| | | *SL-RT* | GTCGTATCCAGTGCAGGGTCCGAGGTATTCGCACTGGATACGACAGGGAT |
| miR166h-3p | | *Frw* | GGCGGTCGGACCAGGCTTC |
| | | *SL-RT* | GTCGTATCCAGTGCAGGGTCCGAGGTATTCGCACTGGATACGACGAGGAA |
| **For fungal DNA quantification** | | | |
| *M. oryzae 28S* | | *Frw* | TACGAGAGGAACCGCTCATTCAGATAATTA |
| | | *Rev* | TCAGCAGATCGTAACGATAAAGCTACTC |
| *F. fujikuroi 28S* | | *Frw* | GAGGCGCGGTGTCGGTGTGCTTG |
| | | *Rev* | CTCTCATATACCCTCCG |
| **For 5' RACE** | | | |
| *EIN2* | | *Rev* | ACCTATCCGGGATGTTGCTGCGATCTGT |
| | | *Rev-Nested* | TCCCATGGTAATCAAAGAGATGCCCCCAGAA |
| *Hox32* | | *Rev* | CCCCCCTCACATGGGCGGATCAAATAGCCGC |
| | | *Rev-Nested* | CCGCCAAAGTTGTCGGTGCATAAGTCTGCA |
| 5' RACE kit Fw primers | | *Fw* | CGACTGGAGCACGAGGACACTGA |
| | | *Fw-Nested* | GGACACTGACATGGACTGAAGGAGTA |
| **For miR166k-166h overexpression** | | | |
| miR166k-166h | | *Frw* | GGGGACAAGTTTGTACAAAAAAGCAGGCTGGTGGCTTGTGGGGAATGTT |
| | | *Rev* | GGGGACCACTTTGTACAAGAAAGCTGGGTTAAAGGGATTGAAGCCTGGT |
| **For *Nicothiana benthamiana* agroinfiltration analysis** | | | |
| *N. benthamiana ubiquitin* | | *Frw* | TCCAGGACAAGGAGGGTATCC |
| | | *Rev* | TAGTCAGCCAAGGTCCTTCCAT |
| miR166k-5p probe | | | CCTCGAGCCAGACAACAAACC |
| U6 | | | TCATCCTTGCGCAGGGGCCA |
| **For polycistronic miRNA genotyping** | | | |
| M0110144 | | *Frw* | CCGGGCAGTGGATTGCGTTGT |
| | | *Rev* | CAAATACTACCAAATATAAGTCACCGG |
| T-DNA LB | | *Frw* | ACTCATGGCGATCTCTTACC |
| **For transgene copy insertions** | | | |
| *Sucrose phosphate synthase* | *LOC_Os02g09170* | *Frw* | TTGCGCCTGAACGGATAT |
| | | *Rev* | CATCCCGAAAAGATCAACCG |
| *Hygromycin* | | *Frw* | CTATTTCTTTGCCCTCGGACGA |
| | | *Rev* | CTTCTACACAGCCATCGGTCC |

**Supplementary Table S2.** T-DNA copy number in *miR166k-166h* plants. The T-DNA copy number was determined by qPCR with the *sucrose phosphate synthase* (*SPS*) gene used as the endogenous reference gene (Ding *et al.*, 2004).

| **Line** | **Number of copies** | **sd** |
|---|---|---|
| M0110144(*miR166k-166h-Ac)* | 1,061914 | 0,096 |

**Supplementary Table S3**. *Cis*-motifs identified in the *MIR166k-166h* promoter.

| Location | Signal Sequence | Name | Description |
|---|---|---|---|
| 63 (-) | YTGTCWC | SEBFCONSSTPR10A | Binding site of SEBF gene found in promoter of PR-10a |
| 145 (+) | TGACY | WBOXNTERF3 | W-box in the promoter region of a ERF3 gene. Related to wounding |
| 171 (+) | TGAC | WRKY71OS | W-box in the promoter region of a ERF3 gene. Related to wounding |
| 211 (-) | AGATCCAA | AGMOTIFNTMYB2 | AG-motif found in promoter of NtMyb2 gene,regulator of the defence-related gene PAL, which is induced by various stress such as wounding or elicitor treatment |
| 346 (-) | CCGTCC | PAL | Consensus of the putative "core" sequences of box-L-like sequences in carrot PAL1 promoter region |
| 432 (-) | TTGAC | WBOXATNPR1 | W-box found in the promoter region of the CAD1-A (cotton (+) delta-cadinene synthase-A) gene. Binding-site of GaWRKY1; GaWRKY1 regulates sesquiterpene biosynthesis in cotton |
| 492 (+) | TGACY | WBOXNTERF3 | W-box in the promoter region of a ERF3 gene. Related to wounding |
| 514 (+) | TGAC | WRKY71OS | W-box in the promoter region of a ERF3 gene. Related to wounding |
| 532 (+) | GAAAAA | GT1-SCAM4 | GT-1 motif found in the promoter of soybean CaM isoform, SCaM-4. Plays a role in pathogen- and salt-induced SCaM-4 gene expression |
| 626 (-) | TGACY | WBOXNTERF3 | W-box in the promoter region of a ERF3 gene. Related to wounding |
| 759 (-) | AWTTCAAA | ERELEE4 | Ethylene responsive element |
| 776 (-) | TTGAC | WBOXATNPR1 | W-box found in the promoter region of the CAD1-A (cotton (+) delta-cadinene synthase-A) gene. Binding-site of GaWRKY1; GaWRKY1 regulates sesquiterpene biosynthesis in cotton |
| 894 (-) | TTGACC | ERE | W-box found in promoter of Arabidopsis thaliana NPR1 gene. Recognized specifically by salicylic acid (SA)-induced WRKY DNA binding proteins and PRms (Pathogenesis-Related maize seed) genes. |
| 912 (-) | TGACY | WBOXNTERF3 | W-box in the promoter region of a ERF3 gene. Related to wounding |
| 984 (-) | TTGAC | WBOXATNPR1 | W-box found in the promoter region of the CAD1-A (cotton (+) delta-cadinene synthase-A) gene. Binding-site of GaWRKY1; GaWRKY1 regulates sesquiterpene biosynthesis in cotton |
| 1031 (-) | [illegible]YYMMCMAMCMM | PAL | Consensus of the putative "core" sequences of box-L-like sequences in carrot PAL1 promoter region |
| 1096 (-) | TGACY | WBOXNTERF3 | W-box in the promoter region of a ERF3 gene. Related to wounding |
| 1228 (+) | GAAAAA | GT1-SCAM4 | GT-1 motif found in the promoter of soybean CaM isoform, SCaM-4. Plays a role in pathogen- and salt-induced SCaM-4 gene expression |
| 1258 (+) | AACGTG | T/G BOXPIN2 | T/G-box found in tomato proteinase inhibitor II (pin2) and leucine aminopeptidase (LAP) genes. Involved in jasmonate (JA) induction of these genes. bHLH-Leu zipper JAMYC2 and JAMYC10 proteins specifically recognaize this motif |
| 1333 (+) | TGACY | WBOXNTERF3 | W-box in the promoter region of a ERF3 gene. Related to wounding |
| 1363 (+) | TGACY | WBOXNTERF3 | W-box in the promoter region of a ERF3 gene. Related to wounding |
| 1388 (-) | GAAAAA | GT1-SCAM4 | GT-1 motif found in the promoter of soybean CaM isoform, SCaM-4. Plays a role in pathogen- and salt-induced SCaM-4 gene expression |
| 1413 (+) | TGACY | WBOXNTERF3 | W-box in the promoter region of a ERF3 gene. Related to wounding |
| 1439 (+) | TGACG | ASF1 | ASF-1 binding-site in CaMV 35S promoter. TGACG motifs are found in many promoters and are involved in transcriptional activation of several genes by auxin and/or salicylic acid |
| 1472 (-) | TTGACC | ERE | W-box found in promoter of Arabidopsis thaliana NPR1 gene. Recognized specifically by salicylic acid (SA)-induced WRKY DNA binding proteins and PRms genes. |
| 1483 (-) | TGAC | WRKY71OS | W-box in the promoter region of a ERF3 gene. Related to wounding |
| 1508 (+) | CCGTCC | PAL | Consensus of the putative "core" sequences of box-L-like sequences in carrot PAL1 promoter region |
| 1552 (+) | TTGACC | ERE | W-box found in promoter of Arabidopsis thaliana NPR1 gene. Recognized specifically by salicylic acid (SA)-induced WRKY DNA binding proteins and PRms genes. |
| 1555 (+) | CCGTCC | PAL | Consensus of the putative "core" sequences of box-L-like sequences in carrot PAL1 promoter region |
| 1599 (-) | TGACY | WBOXNTERF3 | W-box in the promoter region of a ERF3 gene. Related to wounding |
